# Spirituality and Health

**DOI:** 10.1155/2014/682817

**Published:** 2014-01-30

**Authors:** Arndt Büssing, Klaus Baumann, Niels Christian Hvidt, Harold G. Koenig, Christina M. Puchalski, John Swinton

**Affiliations:** ^1^Quality of Life, Spirituality and Coping, Institute of Integrative Medicine, Witten/Herdecke University, 58313 Herdecke, Germany; ^2^Freiburg Institute for Advanced Studies (FRIAS), Albert Ludwigs University of Freiburg, 79104 Freiburg, Germany; ^3^Caritas Sciences and Christian Social Work, Faculty of Theology, Albert Ludwigs University Freiburg, 79098 Freiburg, Germany; ^4^Research Unit of Health, Man and Society, Institute of Public Health, Southern Denmark University, 5000 Odense, Denmark; ^5^Palliative Care, Ludwig Maximilian University of Munich, 81377 Munich, Germany; ^6^Psychiatry & Behavioral Sciences, Duke University, Durham, NC 27710, USA; ^7^Medicine, King Abdulaziz University, 22254 Jeddah, Saudi Arabia; ^8^Medicine and Health Sciences, George Washington Institute for Spirituality & Health, George Washington University School of Medicine & Health Sciences, Washington, DC 20036, USA; ^9^Practical Theology & Pastoral Care, School of Divinity, History & Philosophy, King's College University of Aberdeen, Aberdeen AB24 3UB, UK

Research in the field of mind-body medicine focuses on the complex interaction of psychoemotional, social, spiritual, experiential, and behavioral elements and their impact on health and the handling of disease. Specific approaches intend to investigate and promote patients' own abilities and resources to manage their respective stressors, that is, coping strategies, relaxation techniques, mindfulness meditation, yoga, rituals, prayer, spirituality, and religiosity. An increasing number of published studies have examined the connection between spirituality/religiosity, health, and quality of life. However, the impact of a person's religiosity/spirituality on health is multifaceted and is fraught with methodological controversy since one has to deal with cognitive approaches (specific attitudes and beliefs), emotions, practices (spiritual/religious and secular forms), specific behaviors, reactive strategies to deal with illness (coping), and spirituality/religiosity-based interventions (i.e., meditation, mindfulness, and prayer). Because of this complexity, an interdisciplinary perspective is required for research as well as clinical care.

We would broadly define spirituality as all attempts to find meaning, purpose, and hope in relation to the sacred or significant (which may have a secular, religious, philosophical, humanist, or personal dimension). In particular, spirituality and spiritual practices have commitment to values, beliefs, practices, or philosophies which may have an impact on patients' cognition, emotion, and behavior. Thus, personal spirituality in this sense may influence patients' sense of coherence and their ability to cope with stress, loss, and illness. Spirituality can also have an influence on patients' health behaviors and healthcare decision making, and it can be critically enabling people to reframe their situation. Spirituality can also affect how people relate to meaningful others (i.e., friends, family, and health professionals) who may be significant in their lives. Spirituality can also include people's understanding of the role and importance of transcendence in their lives; however, they may define the term.

This special issue enlisted experts from different disciplines to contribute to new research on the growing body of evidence that spirituality/religiosity impacts health and illness. However, we are aware of the fact that many questions still remain unaddressed and encourage future research.

*Arndt Büssing* *Arndt Büssing* *Klaus Baumann* *Klaus Baumann* *Niels Christian Hvidt* *Niels Christian Hvidt* *Harold G. Koenig* *Harold G. Koenig* *Christina M. Puchalski* *Christina M. Puchalski* *John Swinton* *John Swinton*

